# Indents

**Published:** 1910-04-15

**Authors:** 


### INDENTS.

☞**Brief Articles of Technical or Scientific Value will receive notice and comment. Contributions invited.**

*Aphthous Ulcers of the Mouth.* Touch the ulcer with a pencil of silver nitrate, and immediately after with a pencil of pure zinc. A black coating of colloidal silver is at once formed. This falls off in from twenty-four to thirty-six hours, leaving the ulcer cured.—Dr. Fargin-Fayolle, *La Laboratoire,* May 30, 1909.

*Electric Power from Solar Heat.* Efforts at "harnessing the sun's rays" have hitherto been limited to using them to generate steam in a boiler and run an engine, as in Ericsson's motor and similar devices. Such engines are now in practical use, but have never been widely popular. Now comes Mr. George S. Cove, of Somerville, Mass., who asserts that he can generate useful electric power by using the sun's heat to operate a new form of thermo-electric generator. Such generators are well known and are usually run with the aid of heat from gas flames, but they furnish a current of no great strength. The production of thermo-electricity, as is well known, depends on the fact that in a circuit of two different metals a current may be made to flow, simply by maintaining the two junction-points at different temperatures. Mr. Cove heats one of his junctions with the solar rays. Rene Homer describes the device as follows in *Modern Electrics* (New York):

"The apparatus consists of a little metallic frame which looks like an exaggerated window. The frame contains a number of panes of violet glass, behind which are set, through an asphalt-compound backing, many little metal plugs. One end of these plugs is always exposed to the sunlight, while the other end is cool and sheltered. The invisible rays of the solar spectrum and the invisible ultra-violet rays, after passing through the violet glass, set up a reaction in the peculiar metal alloy used, which produces a continual flow of electrical current into storage batteries.

"The apparatus is automatic, there being a circuit-breaker to sever connections between the separator and storage battery whenever the sun is not shining, and start automatically whenever the sun appears. The apparatus is not affected by weather conditions, and a few clear days suffice to store enough electricity to do away with any possibility of interruption in the service on cloudy days. Ten hours' exposure of the type now being experimented upon will produce enough power to light thirty large tungsten lamps for three days. It is only necessary to increase the size of the generator in order to store enough electricity in a few hours of sunshine to furnish light for a week or more.

"One form of generator shows a voltage of 500 per ten square feet, although there is but a slight flow of current. The generator which has attracted so much attention contains 1,804 plugs, which although individually quite feeble, develop together 60 watts: 6 amperes at 10 volts.

"It is not too much to say that Mr. Cove has revolutionized our conceptions of power generation. Already we can picture the liner of the future propelled by invisible current stored in batteries by the Sun Electrical Generator on a far-away desert and fed into the hold of the vessel in much the same manner as the cartridge belt is fed into a machine gun. The railroad-train of to-morrow, instead of taking on coal and water, will 'plug' into the power-house at the terminal station and pump out enough electricity to make the trip from New York to Chicago. The aeroplane of the future will dart hither and thither, her motors driven by electric energy transmitted by wireless from some far-away Sun Electric power-plant. But best of all is the part it will play in the life of the masses, bringing them cheap light, heat, and power, and freeing the multitude from the constant struggle for bread."

It will be noted from the above that the inventor, besides his thermo-electric effect, which is understandable, claims to produce an additional effect due in some way to the ultra-violet rays and not at present known to science. The technical papers have as yet given no space to Mr. Cove's device, but if his claims for it are justified, a good deal will be said and done about it in the future. However this may be, it is worth notice as the first attempt to use solar heat in a thermo-generator.—*Literary Digest.*

*Dropper-Ampoules: The Latest Device for Chlorform Narcosis.*

Experiments with the newly devised *Dropper-Ampoules* have been conducted at St. Mary's Hospital during the past year. Previously many devices had been tried and found wanting; this one, however, has proved to be the most perfect so far devised for the administration of chloroform by the drop method. This may seem a strong commendation, but its perfection is based upon its simplicity, economy, convenience and safety.

The sealed package containing a sufficient amount of chloroform for one operation has been in vogue for some time. This new device was the result of inventive genius set to task of perfecting the sealed ampoule into a dropper. With the *Dropper-Ampoule* it is almost impossible to saturate the patient because the drop is one-half the size of that which issues from other droppers and the outflow is more readily controlled. It must not be imagined that a small drop means a retardation in the onset of the anesthesia. A small and frequent drop enables the anesthetist to narcotize the patient more rapidly and with a greater degree of safety. Children and the aged are always fearsome subjects for narcosis, but both of these extremes of life accept chloroform safely when narcosis is induced by means of a minute drop. In several operations on a little colored boy anesthesia supervened in one and one-half minutes without struggling, and about three drachms sufficed for the narcosis necessary for the removal of tuberculous glands. This patient was anesthetized three times and was not nauseated once. Three successive operations lasting, conjointly, over two hours, were conducted under a narcosis which did not require all the contents of one ampoule. In private practice such economy could not be secured, but even counting the chloroform left in the container as sheer loss, still the amount used would be much less than by any other method. Moreover, a safe narcosis is always the paramount issue. By requiring the use of a fresh container in each operation the *Dropper-Ampoules* obviate the chance formation of poisonous by-products in the chloroform. The custom of using a pound bottle, indifferently corked and left standing around the operating room until wanted, is to be condemned. Regarding the convenience of this over other devices, a personal trial will convince the most skeptical.—*N. Y. Medical Journal.*

